# Efficacy of exposure and response prevention therapy in mixed reality for patients with obsessive-compulsive disorder: study protocol for a randomized controlled trial

**DOI:** 10.1186/s40359-023-01116-3

**Published:** 2023-04-13

**Authors:** Luzie Lohse, Lena Jelinek, Steffen Moritz, Jannik Blömer, Lara Bücker, Franziska Miegel

**Affiliations:** Department of Psychiatry and Psychotherapy, University Medical Center Hamburg-Eppendorf, Martinistrasse 52, 20246 Hamburg, Germany

**Keywords:** CBT, Psychotherapy, OCD, Exposure therapy, Mixed reality, Augmented reality

## Abstract

Many patients with obsessive-compulsive disorder (OCD) do not receive cognitive behavioral therapy with exposure and response prevention (first line treatment for OCD), for example, due to patients’ fear of the exposure and reservations of the therapists. Technology-supported exposure (e.g., exposure therapy with response prevention in mixed reality [MERP]) for patients with OCD may help to overcome this obstacle. Building upon findings of our pilot study objectives of this study are to evaluate the efficacy, expectations of treatment success, feasibility, and acceptance of MERP as well as to identify possible limitations. In total, 64 outpatients with contamination-related OCD will be recruited and randomized to one of two conditions: MERP (six sessions in six weeks) and self-guided exposure therapy (six exercises in six weeks). Participants will be assessed before (baseline), after the six-week intervention period (post), as well as three months after post assessment (follow-up) regarding symptomatology (Yale-Brown Obsessive Compulsive Scale; Y-BOCS), their subjective evaluation of MERP (acceptance) and sense of presence. The planned study is the first to investigate MERP in patients with OCD.

**Supplementary Information:**

The online version contains supplementary material available at 10.1186/s40359-023-01116-3.

## Trial registration

German Registry for Clinical Studies (DRKS00020969), 14.06.2021.

**Supplementary Information:**

The online version contains supplementary material available at 10.1186/s40359-023-01116-3.

## Background

Obsessive-compulsive disorder (OCD) is a mental disorder characterized by intrusive thoughts (obsessions), and ritualized, repetitive behavior (compulsions) that aim to reduce negative feelings [2, 3]. It has a lifetime prevalence of 1–3% [4, 5], and often takes a chronic course if untreated [6]. Cognitive behavioral therapy (CBT) with exposure and response prevention (ERP) is the first line treatment for OCD, and is recommended by national and international guidelines [7]. In ERP the individual is exposed to stimuli, which trigger obsessions, compulsions or avoidance behavior. The goal of ERP is to confront feared objects or situations, without performing compulsions or avoiding the stimuli.

 Although ERP is highly effective, it is often not [8, 9] or incorrectly [10, 11] applied. Furthermore, only every third therapist or even less uses ERP *in vivo *[9, 12] and less than one third of those who practice ERP *in vivo* apply it according to the guidelines [12], leading to a serious treatment gap – the difference between the prevalence of a disorder and the treated proportion of affected individuals. A large longitudinal study conducted in the United States offers insight into the prevailing treatment gap. In total, 59% of patients with OCD were recommended CBT but only 44% received it [13]. According to a review from Kohn et al. [14] the treatment gap (i.e., untreated patients) in the United Kingdom is estimated between 60% and 68% and in the United States and Germany it is estimated at 59%, contributing to a rather low remission after treatment rate of 46.3% in Germany [15].

There are three main categories of treatment barriers concerning the treatment itself: the patient, the therapist and circumstances. While patients mainly fear being confronted with triggering stimuli during ERP [12], there are multiple treatment barriers on the therapists side, which impede the application of ERP. First, conducting ERP requires additional time (e.g., travelling to patients’ home), and therapists dread cancelled appointments [12] or terminated therapy [16–18]. Second, negative beliefs about ERP (e.g., harming the patient [19–23]) may discourage the therapist to conduct ERP. This dynamic often promotes hesitations to the application of ERP in the first place. Lastly, there exist other barriers such as the COVID-19 pandemic, which encourages social distance and hygiene, and thereby discourages ERP due to a possible risk of infection [24]. Guideline-based treatment therefore is often not or inadequately applied [15] and there is room for improvement. In order to increase the (correct) implementation of ERP and its initiation, we need to address the obstacles associated with its use. One option to increase the acceptance and feasibility of ERP may be the use of technical supported ERP. Here, the structure, mechanisms of ERP and potentially the high effects may be maintained.

### Technical supported exposure response prevention therapy

Technical supported therapy can be used to facilitate the application of ERP as it can address major treatment barriers of ERP *in vivo*: acceptability of treatment [25], a high variety of stimuli as well as improved control over the stimuli. Most studies have been conducted with virtual reality (VR) for anxiety disorders (for an overview, see Carl et al. [26]), for example, specific phobias [26, 27].

### Exposure therapy in virtual reality

Through VR glasses, the individual enters a virtual environment. In exposure therapy in VR (VRET) the individual is confronted with virtual three-dimensional presentations of triggering stimuli. This approach has been extensively studied in anxiety disorders, with at least similar positive effects when compared to exposure therapy *in vivo*. One meta analysis even found a small effect for VRET over *in vivo* (Cohen’s *d* = .35; [28]). Other meta analyses found similar effects [25, 26, 29, 30] for most anxiety disorders except for social anxiety disorder. Moreover, an early study [31] using VR for exposure therapy found that anxiety – measured with subjective units of distress (SUD) – followed the same curve of increase and decrease, such as in a study with fear of flying [32], but the overall anxiety level was less intense in VR. Further, emotions like anger and disgust – measured before and after the intervention – elicited by *in virtuo* and *in vivo *conditions, were not significantly different [33]. While the induction of fear seems similar, previous studies suggest that VRET is generally more accepted in anxiety disorders in contrast to exposure therapy *in vivo* [34, 35], whether this is also true for patients with OCD is still unclear.

### Exposure and response prevention therapy in virtual reality for obsessive-compulsive disorder

In OCD, research on the use of VR for ERP (VERP) is comparatively scarce, potentially due to its heterogeneity of symptoms (e.g., checking-, contamination- or sexual obsessions). A systematic review and recent meta-analysis on symptom and emotional response provocation using multiple kinds of VR-technology, mostly outside the context of ERP, however, supports that VR environments are generally able to provoke obsessive compulsive (OC) symptoms such as anxiety, disgust and the urge to wash in individuals with OCD compared to healthy controls [36], which argues for the feasibility of VERP. This is supported by findings from Miegel, Bücker et al. [37], who demonstrated that arousal due to disgust (measured with SUD) can be induced in VERP in patients with contamination-related OCD. So far, research on VERP for patients with OCD focused on investigating feasibility, evoking fear of contamination [38] and observing checking behavior [39–42]. Preliminary findings of VERP suggest feasibility [43, 44] and potential treatment efficacy [45], [38] and [37]. One recent study [46] investigated VRET in patients with contamination anxiety in an RCT comparing VRET to ERP* in vivo *with each 12 sessions and found a large effect in the reduction of OC symptoms (η_*p*_^*2*^ = .82). As findings stem from small or/and non-clinical samples, they need to be replicated in a representative randomized controlled trial (RCT). Moreover, Miegel, Bücker et al. [37] reported that sense of presence, which represents how present a person feels in a digital augmented environment, was only moderate in their VR environment, which was potentially due to unrealistic movement (a controller was used for teleportation).

### Exposure and response prevention therapy in augmented reality

Augmented reality (AR) glasses (e.g., Magic Leap 2) are much smaller and lighter compared to VR glasses. While looking through the glasses, individuals see their entire surrounding plus additional virtual objects that are projected into the real world. In AR, digital objects are anchored in the physical world, meaning, the digital and the real world are blended. In other words, the objects are “locked” in space as if they were a natural part of the real environment. Thus, the whole experience in AR may seem more real and the individual experiences a higher sense of presence, compared to VR. Also, patients can still see the therapist during ERP in AR, which might promote equally strong therapeutic relationships compared to ERP *in vivo* predicting a more favorable treatment outcome [47] and adherence [48]. AR has already been successfully integrated into medical training [49] and surgery simulation [50] and can be further used to guide implant placement in surgery [51].

By using AR instead of VR, realism and sense of presence could be enhanced. Realism describes the constructive process behind the creation of a person’s own current reality, which is a crucial element of sense of presence. In AR, sense of presence might be improved since the individual moves naturally instead of using a controller. As sense of presence determines how real a person feels in the virtual world, it may impact whether anxiety and disgust can be induced at a personal level and further determine the therapy outcome. Anxiety was able to be induced in AR in healthy individuals measured with self-reported and physiological parameter [52], though less studies are present for OCD. A recent study found that disgust can be induced in patients with OCD in AR and levels of sense of presence were positively related with higher level of anxiety [53]. The idea is based on the notion that anxiety and disgust needs to be inducible, as ERP is based on learning adaptive behaviour in response to triggering stimuli including the corresponding emotional response.

### Exposure and response prevention therapy in mixed reality

Mixed reality (MR) describes the spectrum of augmented reality and virtual experiences and is commonly used in interaction with devices such as the HoloLens or the Magic Leap. It allows the user to interact with real and virtual objects, while seeing their own body as well as the real surrounding at the same time. MR glasses use real time spatial mapping for merging real environments with digital objects and interaction with virtual objects without [54] and with a controller, though the stability of this tool without the controller is not yet established.

According to Hu et al. [55], MR has already been successfully integrated into the medical field (e.g., risk-free surgery training [56] and preoperative communication [57]). ERP in MR (MERP) might be especially relevant for the treatment of psychological disorders such as OCD, with its heterogeneous symptomatology. In MR, objects can be easily tailored to patients. The only preliminary evidence of acceptance of MR stems from research on technology acceptance of MR in museums [58], which notes that the technology is easy to use and highly engaging. According to our current knowledge, to date there is just our pilot study that investigates the feasibility and safety of applying MERP within a clinical sample [1]. 

In this study, we included a sample of *N* = 20 inpatients with contamination-related OCD [1]. During the intervention, all patients received standard care on the psychiatric ward and were randomly assigned to an add-on intervention (MERP + standard care) or control group (standard care only). Over the intervention period of three weeks, the intervention group received six sessions, including four sessions of MERP. For a detailed description, see Sect. 2.7.1. The pilot study [1] focused on safety and feasibility, thus was not powered and designed to investigate efficacy (small sample size and add-on design). Overall, the pilot study demonstrated safety and feasibility of MERP with no significant symptom deterioration in the intervention group. However, results on sense of presence and acceptance were mixed indicating that technical improvements were necessary. Improvement in the quality of the 3D graphics and the possibility of interaction in digital augmented realities are regarded as crucial for implementing a high sense of presence. We performed the necessary technical adjustments also incorporating the feedback of the therapists to investigate not only acceptance and feasibility but also the efficacy in the present RCT. Based on patients’ feedback, technical improvements were implemented and the list of virtual contamination-related objects has been extended (e.g., spray bottle, glass splinter). The planned RCT will be conducted with outpatients in order to be able to draw more robust conclusions.

The aim of the planned RCT is to test the efficacy of MERP in comparison to an active control group with regard to OCD symptomatology (Yale-Brown Obsessive Compulsive Scale [Y-BOCS; [59]), expectations of treatment success (expectations of the process and effectiveness of MERP) and acceptance of treatment (subjective appraisal rating), and feasibility (Temple Presence Questionnaire; TPI; [60]) of the revised MERP intervention. We hypothesize a larger reduction in both the primary outcome (OC symptom reduction according to the Y-BOCS) and secondary outcomes followed by high positive expectation ratings (> 75%) in both groups and subjective appraisal ratings (> 75%) in favor of the experimental group (MERP), in comparison to the active control group (self-guided application of ERP). Further, we postulate a larger decrease in depression (Beck Depression Inventory; BDI-II [61]), anxiety (Generalized Anxiety Disorder Scale-7; GAD-7 [62]), unwillingness to remain in contact with distressing emotions (Brief Experiential Avoidance Questionnaire; BEAQ [63]), state anxiety and distress (measured during MERP: distress ratings of the patient with Subjective Units of Distress; SUD; [64] and physiopsychological parameter: heart rate [puls], gaze fixation [eye-tracking] and skin conductivity [galvanic skin response]). Additionally, we expect symptom improvement from session one to session six in a variety of questions concerning multiple state sensitive dimensions of mental health (insession questionnaire, see supplementary material *C*). Further, we expect larger improvement in quality of life (WHOQoL-BREF global item) compared to the control group. To test these hypotheses, we plan to conduct an RCT with two parallel groups, both receiving active treatment (MERP or self-guided exposure).

## Methods

### Design

The present RCT will be assessor-blinded with parallel assignment to the MERP group and the active control group (self-guided exposure therapy, see German Registry for Clinical Studies [DRKS00020969]). The study includes a baseline and post assessment that takes place in person, a follow-up assessment by phone and an additional online survey, which participants complete at home before each in-person assessment and the telephone interview at follow-up. Participants will be randomized immediately after the baseline assessment. We offer a compensation of 50 Euros (total) for participitation in all three assessments. For an overview, see Table 1.

**Table 1:** Standard protocol items: recommendation for interventional trials (SPIRIT) timeline

		Study Period				
	Enrolment	Allocation	Intervention period	Assessments		
TIMEPOINT**	*-t*_*0*_	0	*6 weeks*	*t*_*0*_	*t*_*1*_	*t*_*2*_
**ENROLLMENT**:						
***Eligibility screen***	X					
***Informed consent***				X		
***Primary measures***				X	X	X
***Secondary measures***				X	X	X
***Allocation***		X				
**INTERVENTIONS**:	MERP or self-guided exposure therapy					
***Intervention***			X			
***Self-guided exposure therapy***			X			
**ASSESSMENTS**:						
***Demographic interview***				X		
***M.I.N.I.***				X		
***Y-BOCS***				X	X	X
***Expectation of MERP***				X	X	
***Subjective Appraisal***					X	
***OCI-R***				X	X	X
***BDI-II***				X	X	X
***WHOQOL-BREF***				X	X	X
***GAD-7***				X	X	X
***TPI***					X	
***BEAQ***				X	X	X
***State sensitive questionnaire***			X			
***Further assessments during MERP (violation of expectancy and psychophysiological parameter) ***			X			
***SUD***			X			

M.I.N.I. = *Mini-International Neuropsychiatric Interview*, Y-BOCS = *Yale-Brown Obsessive Compulsive Scale*, OCI-R = *Obsessive Compulsive Inventory-Revised*, BDI-II = *Beck Depression Inventory*, WHOQOL-BREF = *World Health Organization Quality of Life-BREF (quality of life)*, GAD = *Generalized Anxiety Scale 7*, TPI = *Temple Presence Inventory*, BEAQ *= Brief Experiential Avoidance Questionnaire*, SUD *= Subjective Units of Distress*

### Sample size

No evidence is available for effects of MERP in contrast to an active control group without therapeutic guidance. Thus, we could only calculate sample size approximately. We expect a large effect based on a meta-analysis investigating VRET in anxiety disorders, which found a medium to large effect of VRET compared to an active psychological control condition (*Hedges g* = 0.80; [26]). We expect the effect of MERP to be even higher than in VERP since our technique is more advanced and may induce higher sense of presence, which may have a positive effect on mechanisms like arousal induction [36, 65, 66].

The power analysis (calculated with G*power; [67]) determined a sample size of 64 participants to detect a large effect (*f* = 0.4, α = .05, power of .80).

### Recruitment

Participants will be recruited using contact forms from previous studies of our working group and through advertisement (e.g., newspaper articles, OCD support groups; leaflets; network of therapists; Google AdWords; Facebook and Instagram). A landing page (www.uke.de/zwang_mr) and a flyer will be used for recruitment.

### Inclusion and exclusion criteria

We will recruit outpatients with OCD. The diagnosis of OCD will be confirmed by the Mini-International Neuropsychiatric Interview 7.0.2 (M.I.N.I.: [68]; German version: [69]), that is based on the Diagnostic and Statistical Manual of Mental Disorders (5th ed.; DSM-5) and the content of OCD (contamination-relation) will be assessed by the Yale-Brown Obsessive-Compulsive Scale; Y-BOCS [59, 70]. Further psychiatric diagnoses for in- and exclusion criteria will be verified with the M.I.N.I. interview. Participants have to fulfill the following criteria:

#### Inclusion criteria

- the presence of contamination-related obsessions and compulsions
- provision of informed consent
- age between 18 and 80 years
- sufficient command of the German language
- willingness to participate in MERP or self-guided exposure for six weeks

#### Exclusion criteria

- a past or present diagnosis of schizophrenia or bipolar disorder
- a present severe substance use disorder
- acute suicidality
- a severe neurological disorder associated with OCD
- current inpatient treatment (including day care treatment)

### Randomization and assessor blindness

The randomization will be administered by the principal investigator by using an online randomization software (https://miniwebtool.com/de/number-randomizer/). The participants will be randomized either to MERP or the self-guided exposure therapy. When a participant enters the study, she/he will receive the consecutive identification number. The participant will be randomized after baseline assessment. All assessors are blinded and will not be informed about group allocation. Unplanned unblinding will be documented.

### Procedure

Patients eligibility will be checked within a telephone interview. Eligible patients will be invited for the baseline in-person interview. The participants receive detailed information about the study and informed consent will be provided by the interviewer. The online survey will be provided before each interview. Demographic information, psychiatric diagnoses (M.I.N.I.) and OC symptoms (Y-BOCS) will be assessed in-person. After the baseline interview (t0), participants will receive an envelope, which includes information about the group allocation. During the following six weeks, participants will receive MERP once a week or conduct self-guided ERP based on a manual they receive shortly after the interview by the principal investigator. Patients will be asked to send back the manual to the office before the post assessment, which will maintain the allocation blinding of the interviewer. Both groups will attend the in-person post assessment after six weeks (post assessment; t1) and a telephone interview three months (follow-up; t2) after the post assessment, whereby the Y-BOCS will be administered again. Before, during and after each session (MERP or self-guided exposure), multiple parameters (e.g., physiopsychological parameter [only in MERP], state sensitive questions [see supplementary material *C*] and SUD) will be assessed.

### Intervention

#### Exposure and response prevention therapy in mixed reality

Well-trained and supervised psychotherapists in post graduate training (master´s degree in psychology) will conduct the MERP. Each session will last between 60 and 90 min. The medical history is taken in the first session. During the second session, psychoeducation will be conveyed and a detailed preparation for MERP will be conducted. Part of the preparation is to rate the difficulty and associated distress of the objects that are available in the MR. The rating will be used for the next MERP and guides the choice of objects (supplementary material *D*). The MERP will start in session three with a medium difficult exposure, which will increase in the level of difficulty during the following sessions.

Before, every three minutes during and after the exposure, the level of distress (SUD) will be rated and saved on a tablet by the therapist. Before and after each session, the state sensitive questionnaire will be filled out by the participant. Additionally, participants will be asked before the exposure about their predicted feared event and after the exposure whether it occurred (i.e., expectation violation). Each session is closed with a positive note and motivation for self-reward.

#### **Technical application of exposure therapy with response prevention in mixed reality**

**Fig. 1:**
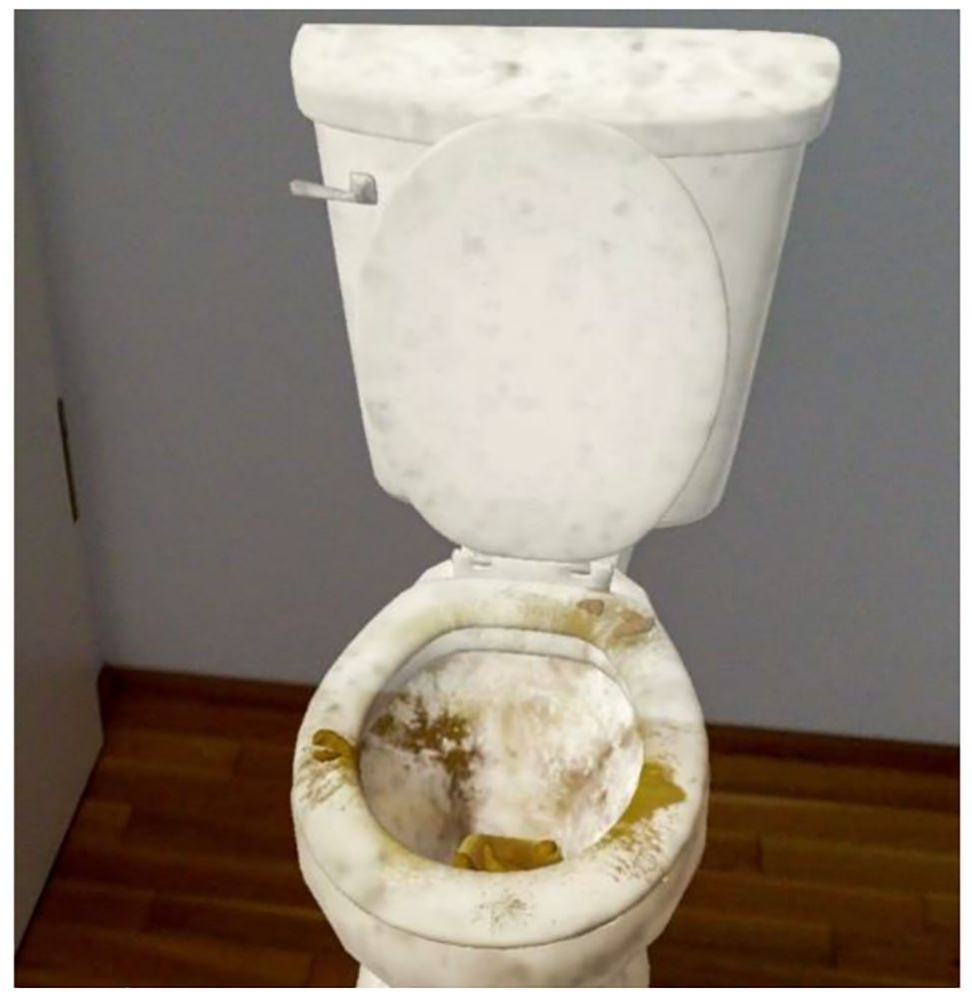
Virtual toilet.

**Fig. 2:**
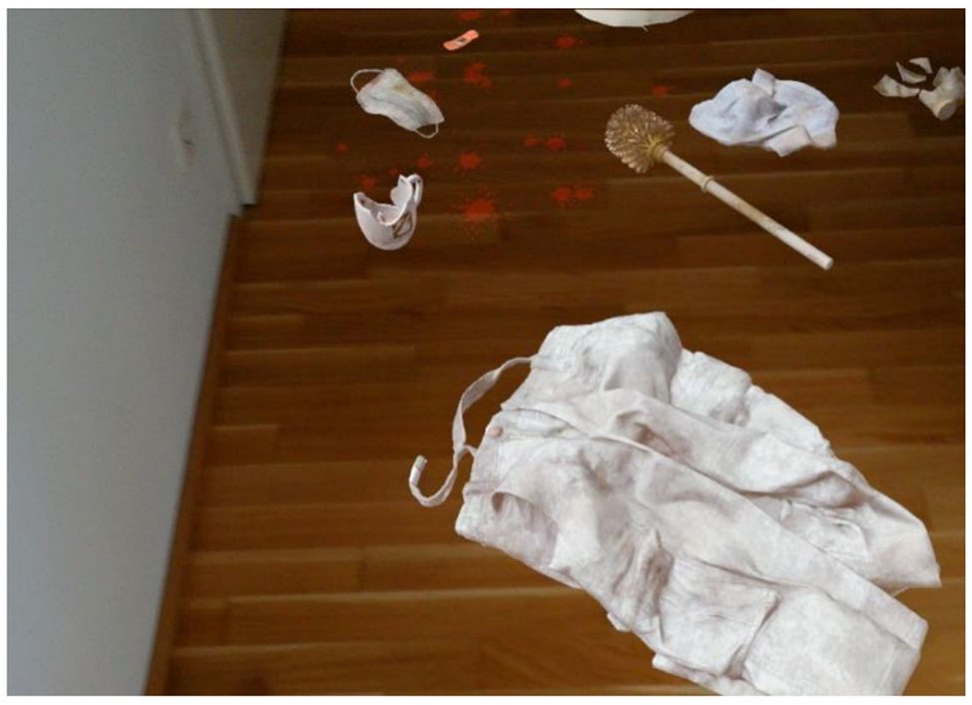
Virtual objects on the floor.

The MERP is conducted with mixed reality glasses (brand: MagicLeap1), which projects virtual objects into the reality – thus – extends the reality. The software is based on *Game Engine Unity* (2020.3.22f1). Before the session, the therapist can select objects (see Figs. 1 and 2; therapist mode) and place them into the room using a controller. Then, the mode will be switched into the patient mode and the objects are locked in space. The controller can be used by the patients to interact with the objects. Through the tablet, an artificial virus scan can be administered (green spots mark objects or places of high contamination) and coughing in three levels of intensity can be switched on and off. When the patient touches objects which are recognizable contaminated (e.g., green spots represent a virus), the particles spread towards the patient’s hands. Physiopsychometric parameters such as gaze fixation (eye-tracking) will be assessed with the MR-glasses and electrodermal skin conductivity (galvanic skin response) will be assessed with a moodmetric ring during MERP.

#### Self-guided exposure and response prevention therapy

The control group will conduct self-guided ERP according to a manual, which is based on the same structure as the therapists’ MERP manual. The manual is divided into six exercises – one exercise for each week. A plane list provides the option to note the selected objects and rate them in terms of expected level of distress on a scale from (0) *no distress* to (100) *extreme distress* for each exercise. A participant will first reflect their medical history, obsessions and compulsions (comparable to session 1 of MERP) before receiving psychoeducation in the next exercise (comparable to session 2 of MERP). During the second exercise, the participants are asked to plan their first exposure for the next exercise (comparable to session 2 of MERP). The participant will choose, plan and document their exposure with the manual (comparable to sessions 3 to 6 of MERP). The level of difficulty is supposed to increase from one exposure to the next one.

### Measures

### Primary measure and secondary measures

#### Yale-brown obsessive compulsive scale (Y-BOCS)

The Y-BOCS (English version [70]; German version [71]) is a semi-structured interview used to assess OC symptom severity. The Y-BOCS consists of two parts: (1) a symptom checklist to identify current as well as former OCD contents, and (2) structured questions designed to determine symptom severity. Each item can be rated on a 4-point Likert scale, ranging from (0) *no symptoms* to (4) *severe symptoms*. The total score ranges from 0 to 40. Ranges of severity are: 0–7 (subclinical), 8–15 (mild), 16–23 (moderate), 24–31 (severe), 32–40 (extreme). The good psychometric properties of the scale have been verified for the German version (high interrater reliability between *r* = .74 – *r* = .97) and good internal consistency (Cronbach´s *α* = 0.80;[72]).

### Secondary outcome measures

#### Obsessive compulsive inventory (OCI-R)

The OCI-R (English version [73]; German version [74]) measures the severity of OC symptoms overall and in six dimensions: checking, contamination, ordering, hoarding, obsessing, and neutralizing [75] with 18 items on a 5-point Likert scale ranging from *(0) not at all* to *(4) extremely strong*. The possible range of scores is between 0 and 72 with a cut-off score of 21 indicating the likely presence of OCD. The original scale as well as the German version (used in the present study) have shown good to excellent psychometric properties (German scale: excellent internal consistency [except for neutralizing], good convergent and divergent validity [74]; English scale: high internal consistency [except for neutralizing] and moderate to high test-retest-reliability [75]) also in a clinical sample [76].

#### Expectations

Expectations about the application and treatment success of MERP will be assessed with an adapted and extended version of the Attitudes Towards Psychological Online Interventions Questionnaire [77] and the Milwaukee Questionnaire [78] (see supplementary material *A*) with a total score between 0 and 64. Expectations of MERP will be rated by both groups before and after (concerning another MERP) their intervention. The scale consists of 16 items (e.g., “*I believe that MERP is helpful for washing obsessions*”) rated on a Likert scale ranging from *(0) completely agree* to *(4) completely disagree* and one open question (“*What do you expect from MERP?*”).

#### Temple presence inventory (TPI)

The TPI [60] consists of 42 items in its original version and measures sense of presence based on eight dimensions of telepresence. The scale has also been used in the pilot study and was previously adapted to match MERP (e.g., one item of the perceptual realism subscale was omitted because it did not fit to the present study). Thus, it consists of three scales, which assess sense of presence: perceptual realism, engagement, and spatial presence with 15 items and is rated on an 8-point Likert scale with higher values indicating a higher sense of presence. The internal consistency of the scale has shown to be good [60].

#### Subjective appraisal rating

The subjective appraisal of MERP and the self-guided exposure therapy will be assessed with a questionnaire based on the scale used by Miegel, Bücker et al. [37], the APOI [77]), and the Technology Usage Inventory (TUI; [79]; supplementary material *B*) – that will be rated on a 5-point Likert scale ranging from *(1) completely agree* to *(5) completely disagree.* An example of an item of the subjective appraisal is “*I would prefer MERP over exposure in vivo*“ and an example of an item of the subjective appraisal of self-guided exposure is „*During the self-guided exposure I missed the therapeutic support*”.

#### Subjective units of distress questionnaire (SUD)

The SUD [64] will be used to assess distress during the ERP sessions. The therapists will ask the patient every three minutes about their current state of distress using a scale ranging from *(0) no distress* to *(100) extreme distress*. Higher scores indicate higher distress.

#### Brief experiential avoidance questionnaire (BEAQ)

The BEAQ [63] assesses experiential avoidance, which describes the unwillingness to remain in contact with distressing emotions. The scale consists of 15 items and is rated on a 6-point Likert scale ranging from *(1) completely agree* to *(6) completely disagree*. The total score is between 15 and 90. A higher score indicates high experiential avoidance. The BEAQ showed good internal reliability (Cronbach´s *α* between .8 [clinical sample] and *.81* [student sample]). It has acceptable to good test-retest reliability between *r = .77* [clinical sample] and *r = .86* [student sample] according to Schaeuffele et al.[63].

#### Quality of life (global item of the quality of life-abbreviated version)

The Quality of Life – abbreviated version (WHOQOL-BREF) assesses quality of life [80] and is a short version of the WHOQOL-100, with 26 items. Only the global item will be used in the planned RCT, which concerns the general quality of life “*How would you rate your quality of life?*”. The question will be rated on a 5-point Likert scale ranging from (1) *very bad* to (5) *very good*. A high score indicates a high quality of life.

#### Beck depression inventory-II (BDI-II)

The BDI-II [81] was conducted in its German version [82] and assesses symptom severity of depression (i.e., affective, cognitive, motivational, vegetative, and psychomotor components). The self-report scale is based on DSM-IV criteria [83] and contains 21-items, which are rated on a 4-point Likert scale with differently marked anchors. The total score ranges from 0 to 63. The total scores can be interpreted as no depressive symptoms (0–8), minimal depressive symptoms (9–13), light depressive symptoms (14–19), medium severe depressive symptoms (20–28), and severe depressive symptoms (29–63). The German version has shown a good internal consistency (Cronbach’s *α* ≥ 0.84), and good test-retest reliability, which exceeded *r* ≥ .75 in nonclinical samples [82].

#### Generalized anxiety disorder scale − 7 (GAD-7)

The GAD-7 [62] is a self-report questionnaire, which assesses generalized anxiety. It consists of 7 items, which are rated on a 4-point Likert scale ranging from (0) *not at all* to (3) *nearly every day*. The possible range of scores is between 0 and 21. Scores indicate mild (5), moderate (10) and severe (15) anxiety. The scale has good reliability, as well as criterion, construct, factorial, and procedural validity [62].

#### State sensitive insession questionnaire

The state sensitive insession questionnaire (supplementary material *C*) assesses how the person feels during the present moment with 20 items on a 5-point Likert scale from *(1) completely agree, (2) agree, (3) not sure, (4) disagree* to *(5) completely disagree.* The first five items are similar to the one used in Miegel et al. [98]. Item 1 and 2 concern meta-cognitive beliefs (e.g., “*It is important that I monitor my thoughts*”). Item 3 to 6 directly assess OC-specific states (e.g., “*I have obsessions right now*”). Item 7 to 12 ask for the emotional state (e.g., “*I am angry*”). Item 13 and 14 assess resistance against obsessions. Item 15 to 16 assess disgust. Item 17 to 18 ask about dizziness and malaise. Item 19 to 20 assess self-competence (e.g., “*I am convinced that I cannot face by obsessions and compulsions*”).

#### Further assessments during MERP

During MERP physiopsychological parameter such as gaze fixation [eye-tracking] and skin conductivity [galvanic skin response] will be assessed. Skin conductivity will be assessed with a mood ring [84], while gaze fixation will be assessed with the MR-glasses. Violation of expectancy will be assessed by different questions before (e.g., “How likely do you think it is that your worst fear actually comes true?”) and after (e.g., “How much did your worst fear come true?”) each MERP. 

### Statistical analyzes

To evaluate the change in OC and comorbid symptoms between MERP and self-guided exposure, analyses of covariance (ANCOVA) are planned with treatment as the between-subject factor (MERP vs. self-guided exposure therapy), the difference in the scores of the outcomes (t0 – t1 and t0 – t2, respectively) as the dependent variable, and the baseline score of each outcome as the covariate. Paired *t*-test will be applied for within group differences from baseline to post and from baseline to follow-up assessment. First, analyzes will be conducted with the complete cases sample (complete participation at t0, t1and t2) and an intention-to-treat analysis will follow (ITT).

Acceptability of MERP will be displayed descriptively by means and standard deviations. Graphs will show frequencies of approval or rejection of all items. To assess safety of MERP the Reliable Change Index (RCI) [85] for the change in the Y-BOCS total score from baseline to post assessment for each patient will be calculated to assess meaningful clinical decline. An RCI above 1.96 (i.e., ~ 5% level) is regarded as significant. The change of expectation of MERP at t0 (before randomization) to t1 will be analzsed with ANCOVAs. Differences in the scores of expectation (t0 – t1) will be chosen as the dependent variable, and the baseline score of treatment expectation as the covariate. Within group differences will be assessed with paired *t*-test from baseline to post assessment.

As insession variables, expectancy violation and habituation (within- and between sessions) will be analyzed. Items of the state sensitive insession questionnaire will be analyzed by linear mixed effect models similar to previous studies [86].

Pearson’s correlation will be calculated for the relationship between sense of presence and OC symptom reduction (Y-BOCS). Further sense of presence, expectation of MERP and acceptance will be displayed graphically.

Effect sizes will be described by Cohen’s *d* (*d* ≈ 0.50 small effect, *d* ≈ 0.80 medium effect, *d* > 0.80 large effect; 98) as well as partial eta square (η_*p*_^*2*^ ≈ 0.01 small effect, η_*p*_^*2*^ ≈ 0.06 medium effect, η_*p*_^*2*^ ≈ 0.14 large effect).

### Ethical standards

The RCT will be conducted in compliance with the Declaration of Helsinki and is approved by the local ethics committee of the center for psychosocial medicine of the University Medical Center Hamburg-Eppendorf, Germany (local psychological ethic committee at the center for psychosocial medicine [LPEK-0216]). The authors declare that all procedures contributing to this study comply with the ethical standards of the relevant national and institutional committees on human experimentation and with the Helsinki Declaration of 1975, as revised in 2008. Furthermore, the authors assert that all procedures contributing to this work comply with the ethical standards of the relevant institutional and national guides on the care and use of laboratory animals. Unexpected adverse events will be documented. The anonymous data will be stored for 10 years, will be used for research only and will be deleted in the case of revocation of the declaration of consent. Protocol amendments will be communicated with study principal, ethic committee and the Hamburgische Investitions- und Förderbank and adjusted in the trial registration.

## Discussion

This is the first larger study, which investigates efficacy, acceptability, expectations and feasibility of MERP. Positive findings will support the use of MERP as an alternative treatment or supplement to ERP *in vivo* for patients with OCD and may stimulate further research with larger RCTs.

More effective treatment options for OCD are still needed, since drop out rates for ERP* in vivo* are high [16–18] and barriers from the side of the patients, the therapists [12, 87] and circumstances [24] are still present. Therapists may be encouraged to broaden their repertoire of application, benefit from local administration in their own office and apply MERP to patients, who are too afraid of confrontation *in vivo*, since the confrontation is “only” in MR and may therefore appear less aversive in the first place. MERP does not aim to replace ERP *in vivo*, though may offer additional insight by tracking pupils and determining otherwise hidden avoiding behaviours.

According to VRET it can be assumed that MERP is also accepted by patients with OCD [25]. Unfortunately, we only have preliminary evidence for MERP from the pilot study [1]. This current study will look at expectations of treatment success of MERP and various domains of acceptance (perceived efficacy, perceived feasibility, perceived risk of drop out), while contrasting self-guided ERP* in vivo*.

ERP is an effective treatment for patients with OCD [88–92], nevertheless, working mechanisms (e.g., expectancy violation and habituation; [93]) and crucial factors of ERP have not been investigated properly in VR [25] nor MR, before. Analyzing those factors will elucidate which ones promote efficacy of treatment and determine the focus of treatment improvements. Sense of presence is widely regarded as such as a central factor [94], and will be part of our analysis. Since research on expectancy violation in OCD is rare [95, 96], its investigation in a controlled setting – likewise provided in MR – is advised.

Further, we will investigate the most common comorbidities (e.g., depression and anxiety disorders [97]), which may hinder the effectiveness of MERP. This potentially will help to adjust the content of MERP for those patients in the future. Additionally, we will investigate the effect of MERP on central comorbidities and clarify whether the intervention has secondary benefits.

### Limitations and future studies

There are possible limitations that need to be discussed. First, only patients who are generally open to ERP reach out to this study. A clear statement whether MERP is an alternative to fearful patients is not possible, since the representativeness of the sample is limited. Nevertheless, we also assess reasons of patients concerning their drop-outs, which may reduce this limitation. Second, we will not be able to transfer findings onto other subtypes of OCD besides contamination-related OCD. Though, future studies may be built upon our findings and may extend the repertoire towards other symptom domains. Third, patients will be able to continue their outpatient therapy including ERP, thus symptomatic improvement due to MERP will be difficult to reveal. However, both groups are equally allowed to continue their treatment and its type and amount will be assessed and considered in the analyzes.

One strength of our study is that both groups receive active treatment, which includes exposure. Therefore, the mode of application will determine the efficacy. Furthermore, our study facilitates a close investigation of efficacy, acceptability, expectations of MERP and emotion induction of MERP. In contrast to VR, the application of MR is rather intuitive and easy to learn for therapists and patients. Thus, we expect that positive findings may promote actual application by therapists.

If proven effective and accepted, this therapy could facilitate a more guideline compliant treatment and future studies should discover the application for patients with different obsessions (e.g., checking). In general, the study facilitates knowledge, which can be used for the improvements of the intervention. Multiple variables will be assessed, which may explain smaller than expected effects, and direct future research.

### Trial status

The first participant was enrolled in March 2022. At present, 30 participants attended their baseline assessment. At the time of submission of this study protocol, participants were still being recruited, and no data had been extracted and analyzed yet. All future changes to the study protocol will be recorded in an independent amendment. SPIRIT guidelines were followed for the whole article.

### Timetable and research plan

The present study will be carried out in 2022 and 2023. Recently, the pilot study ended and findings were used to optimize the software and study setup of this larger study. The recruitment has already started, and 30 participants have been randomized. The recruitment and treatment will continue till the end of 2023. This study protocol was started in June 2022 and will be handed in till spring 2023. During the second half of 2023 the follow-up assessment will be carried out. Thereafter, data will be prepared and analyzed for two additional publications in international journals. Personnel resources were very high at the beginning of the study, thus the study protocol was submitted later.

## Conclusion

This study protocol facilitates study replication for future studies. Since this study is the first of its kind – including a large sample – it will direct future research. If this study can demonstrate that MERP is effective, well accepted with positive expectations of MERP and able to induce negative emotions, larger RCTs are needed. Through this study, we will learn about which factors are crucial for treatment efficacy and need to be considered in future research.

## Electronic supplementary material

Below is the link to the electronic supplementary material.

Supplementary Material 1

## Data Availability

Data will be available upon reasonable request.

## Abbreviations

OCD: obsessive-compulsive disorder
CBT: cognitive behavioral therapy
ERP: exposure and response prevention
RCT: randomized controlled trial
VR: virtual reality
AR: augmented reality
MR: mixed reality
VRET: exposure therapy in virtual reality
VERP: exposure and response prevention therapy in virtual reality
NICE: National Institute for Health and Care Excellence
SUD: subjective units of distress
Y-BOCS: Yale-Brown Obsessive Compulsive Scale
M.I.N.I.: Mini-International Neuropsychiatric Interview
OCI-R: Obsessive Compulsive Inventory-Revised
TPI: Temple Presence Questionnaire
GAD-7: Generalized Anxiety Disorder Scale-7
BEAQ: Brief Experiential Avoidance Questionnaire
BDI-II: Beck Depression Inventory-II
WHOQOL-BREF: World Health Organization Quality of Life-BREF (quality of life)
ITT: intention to treat analysis
EFRE: Europäischer Fond für Regionale Entwicklung
