# Supplementary material for: Efficacy of exposure and response prevention therapy in mixed reality for patients with obsessive-compulsive disorder: study protocol for a randomized controlled trial
Source: BMC Psychol. 2023 Apr 13;11:113. doi: 10.1186/s40359-023-01116-3 (PMC10100604; doi:10.1186/s40359-023-01116-3)
Supplement: Supplementary file 1 — Supplementary Material 1 [file 40359_2023_1116_MOESM1_ESM.docx]

**Supplementary Material**

Supplementary material *A* (Appendices A): Expectation of MERP

|  | **Question** | **Rating** | | | | |
| --- | --- | --- | --- | --- | --- | --- |
| 1 | Have you ever performed an exposure before? | Yes  **Follow-up question**:  Self-guided exposure or exposure guided by a therapist | No | | | |
| 2 | Have you participated in a tech-based (mixed and virtual reality) exposure before? | Yes  More than Once? | No | | | |
| 3 | **Follow-up question**: If yes, in which one? | Exposure therapy in virtual reality | | Exposure therapy in mixed reality |  |  |
|  |  | **Completely agree agree** | | **Agree** | **Disagree** | **Completely disagree** |
| 4 | I believe exposure therapy in mixed reality can be useful in the treatment of mental illness in general. |  | |  |  |  |
| 5 | I think I will come to every session. |  | |  |  |  |
| 6 | I find it easier to face my washing compulsions in exposure therapy in mixed reality than exposure therapy in reality. |  | |  |  |  |
| 7 | I will benefit from therapeutic guidance in exposure in mixed reality. |  | |  |  |  |
| 8 | I am afraid of the experience in mixed reality. |  | |  |  |  |
| 9 | I am not afraid that the technique will cause discomfort (e.g., dizziness) in me. |  | |  |  |  |
| 10 | I am afraid of the exposure itself. |  | |  |  |  |
| 11 | I think that exposure in mixed reality makes me tense. |  | |  |  |  |
| 12 | It will not be difficult for me to actually implement what I have learned exposure therapy in mixed reality in everyday life. |  | |  |  |  |
| 13 | Exposure therapy in mixed reality cannot give me important suggestions to better deal with my washing compulsions. |  | |  |  |  |
| 14 | I think that my therapist will debrief the performance of the exposure exercise with me and give me tips. |  | |  |  |  |
| 15 | I do not expect any longer term effects from my participation in exposure therapy in mixed reality. |  | |  |  |  |
| 16 | I believe that I may discontinue exposure therapy in mixed reality. |  | |  |  |  |
| 17 | I believe exposure therapy in mixed reality can be helpful for washing compulsions. |  | |  |  |  |
| 18 | The therapist will professionally assist me with exposure in mixed reality. |  | |  |  |  |
| 19 | Skills that help me better manage washing compulsions I learn in exposure therapy in reality rather than exposure therapy in mixed reality. |  | |  |  |  |
| 20 | After completing exposure therapy in mixed reality, I will be better able to deal with my washing compulsions. |  | |  |  |  |
| 21 | What do you expect from mixed reality exposure therapy? | | | | | |

Supplementary material B (Appendice *B*): Subjective appraisal scale

|  | | | **Com-pletely disagree** | **Agree** | **Neither true nor false** | **Disagree** | **Comp-letely disagree** |
| --- | --- | --- | --- | --- | --- | --- | --- |
| 1 |  | I expect little long-term effects from MERP/self-directed exposure. |  |  |  |  |  |
| 2 | MR | The virtual objects had a similar effect on my constraints as in reality. |  |  |  |  |  |
| 3 | SG | With self-directed exposure, I like that I have the flexibility to manage my time. |  |  |  |  |  |
| 4 |  | I had to get over myself to participate in MERP/self-directed exposure. |  |  |  |  |  |
| 5 | MR | The virtual objects seemed realistic to me. |  |  |  |  |  |
| 6 | SG | With self-directed exposure, I like that I am independent of location. |  |  |  |  |  |
| 7 | MR | I felt in good hands with the therapist. |  |  |  |  |  |
| 8 | SG | With the self-directed exposure, I lack therapeutic support. |  |  |  |  |  |
| 9 |  | Exposure to MERP/self-directed exposure was able to reduce my obsessive thoughts. |  |  |  |  |  |
| 10 | MR | The items in the exposure in MERP matched my constraints. |  |  |  |  |  |
| 11 | MR | The items in the exposure were relevant to me personally. |  |  |  |  |  |
| 12 | SG | I can easily integrate the self-directed exposure into my daily life. |  |  |  |  |  |
| 13 |  | The application of this MR technology/the application of the manual for self-directed exposure was complicated. |  |  |  |  |  |
| 14 | MR | I think MERP is easier for me to master than exposure in reality. |  |  |  |  |  |
| 15 | SG | I think that self-directed exposure is easier for me to master than exposure under therapeutic supervision. |  |  |  |  |  |
| 16 |  | I found it difficult to actually implement the suggestions from MERP/self-guided exposure in everyday life. |  |  |  |  |  |
| 17 | MR | I am more likely to stay "on the ball" with exposure in reality than with MERP. |  |  |  |  |  |
| 18 | SG | I am more likely to "stay on the ball" with therapeutically guided exposure in reality than with self-directed exposure. |  |  |  |  |  |
| 19 |  | I feel like MERP/self-directed exposure was able to help me. |  |  |  |  |  |
| 20 |  | I consider the treatment principle of MERP/self-directed exposure to be useful in principle. |  |  |  |  |  |
| 21 |  | MERP/self-directed exposure was able to reduce my compulsivity. |  |  |  |  |  |
| 22 | MR | MERP has helped me identify what problems I need to address and solve. |  |  |  |  |  |
| 23 | MR | I think MERP is easier for me to master than exposure in reality. |  |  |  |  |  |
| 24 |  | The use of MR technology/the application of the manual for self-directed exposure was overall straightforward. |  |  |  |  |  |
| 25 |  | MERP/self-directed exposure can be just as effective as exposure in reality under therapeutic supervision. |  |  |  |  |  |
| 26 |  | I avoid less by MERP/self-directed exposure. |  |  |  |  |  |
| 27 |  | I grasped the theoretical therapy concepts worse in MERP/self-guided exposure than in real-life exposure in therapeutic supervision. |  |  |  |  |  |
| 28 |  | In MERP/self-directed exposure, the treatment offered was worse for my individual needs. |  |  |  |  |  |
| 29 |  | MERP/self-directed exposure was able to give me important ideas to better deal with my problems. |  |  |  |  |  |
| 30 |  | The use of MR technology/the application of the manual for self-directed exposure was easy to understand. |  |  |  |  |  |
| 31 | MR | I would prefer exposure in MR to exposure in reality. | Yes | No | No preference | | |
| 32 | SG | I would prefer self-directed exposure to exposure with therapeutic guidance. | Yes | No | No preference | | |
| 33 | MR | What advantage would you see of MERP over therapeutically supervised exposure in reality? |  | | | | |
| 34 | SG | What advantage of self-directed exposure would you see over therapist-guided exposure in reality? |  | | | | |
| 35 | MR | What disadvantage of MERP would you see compared to exposure to reality? |  | | | | |
| 36 | SG | What disadvantage of self-directed exposure would you see over therapist-guided exposure in reality? |  | | | | |
| 37 | MR | What objects were you still missing in MERP to better address your constraints? |  | | | | |

Note: MR*: Items only shown to participants of MERP,* S*G: Items only shown to participants of the self-guided esposure therapy*

| Please rate how you are **currently** feeling, how strongly the following statements apply to you **at this moment**. | **Completely**  **agree** | **Agree** | **Not sure** | **Disagree** | **Com-pletely disagree** |
| --- | --- | --- | --- | --- | --- |
| **1.** It is important that I constantly monitor my thoughts. | -2 | -1 | 0 | +1 | +2 |
| **2.**  I should be in control of my thoughts at all times. | -2 | -1 | 0 | +1 | +2 |
| **3.**  At this moment, I am suffering greatly from obsessive thoughts. | -2 | -1 | 0 | +1 | +2 |
| **4.**  Right now, strong obsessive thoughts are imposing themselves. | -2 | -1 | 0 | +1 | +2 |
| **5.**  At this moment, I am suffering greatly from obsessive-compulsive behavior. | -2 | -1 | 0 | +1 | +2 |
| **6.**  I have a strong urge to perform compulsive actions right now. | -2 | -1 | 0 | +1 | +2 |
| **7.** My mood is poor. | -2 | -1 | 0 | +1 | +2 |
| **8.** I am sad. | -2 | -1 | 0 | +1 | +2 |
| **9.** I am afraid. | -2 | -1 | 0 | +1 | +2 |
| **10.** I am tense. | -2 | -1 | 0 | +1 | +2 |
| **11.** I am angry. | -2 | -1 | 0 | +1 | +2 |
| **12.** I am annoyed. | -2 | -1 | 0 | +1 | +2 |
| **13.**  I'm resisting obsessive thoughts right now. | -2 | -1 | 0 | +1 | +2 |
| **14.**  I am convinced that in the next hour I will manage to resist my obsessive thoughts. | -2 | -1 | 0 | +1 | +2 |
| **15.**  I feel disgust. | -2 | -1 | 0 | +1 | +2 |
| **16.**  At that moment, I feel disgust. | -2 | -1 | 0 | +1 | +2 |
| **17.**  I feel dizziness. | -2 | -1 | 0 | +1 | +2 |
| **18.**  I have a sinking feeling in my stomach. | -2 | -1 | 0 | +1 | +2 |
| **19.**  I am convinced that I cannot face my compulsions. | -2 | -1 | 0 | +1 | +2 |
| **20.**  I am convinced that I cannot endure the anxiety during my compulsions, without performing compulsive actions. | -2 | -1 | 0 | +1 | +2 |

Supplementary material (Appendices *C*): Insession Questionnaire

Supplementary material (Appendice *D*): Objets in the mixed reality; (0) *no arousa*l and (100) *extreme arousal*

| **Objects** |
| --- |
| Dirty Sink |
| Toilet paper roll |
| Maggot |
| Dirty cigarette box |
| Single cigarette end |
| Cockroach |
| Dirty toilet paper |
| Crinkly surgical mask |
| Broken glas |
| Dirty Syringe |
| Dirty plaster |
| Dirty handkerchief |
| Crumpeled handkerchief |
| Fly |
| Dirty trouser |
| Dirty underwear |
| Bloody bandage |
| Broken bloody cup |
| Used condom |
| Hairy scrunchy |
| Slimy Handkerchief |
| Dirty Toilet |
| Urine cup |
| Dirty toilet brush |
| Hairy hairbrush |
| Blood Splatter |
| Dirty spots |
| Spray bottle |
| Chemical spray bottle |
| Visible virus particles |
| Coughing |
